# Artificial Intelligence for Alzheimer’s Disease: Promise or Challenge?

**DOI:** 10.3390/diagnostics11081473

**Published:** 2021-08-14

**Authors:** Carlo Fabrizio, Andrea Termine, Carlo Caltagirone, Giulia Sancesario

**Affiliations:** 1 Laboratory of Experimental and Behavioral Neurophysiology, IRCCS Santa Lucia Foundation, 00143 Rome, Italy; c.fabrizio@hsantalucia.it (C.F.); a.termine@hsantalucia.it (A.T.); 2 Department of Clinical and Behavioral Neurology, IRCCS Santa Lucia Foundation, 00179 Rome, Italy; c.caltagirone@hsantalucia.it; 3 Biobank, IRCCS Santa Lucia Foundation, 00179 Rome, Italy; 4 European Center for Brain Research, Experimental Neuroscience, 00143 Rome, Italy

**Keywords:** Alzheimer’s disease, diagnosis, machine learning, artificial intelligence

## Abstract

Decades of experimental and clinical research have contributed to unraveling many mechanisms in the pathogenesis of Alzheimer’s disease (AD), but the puzzle is still incomplete. Although we can suppose that there is no complete set of puzzle pieces, the recent growth of open data-sharing initiatives collecting lifestyle, clinical, and biological data from AD patients has provided a potentially unlimited amount of information about the disease, far exceeding the human ability to make sense of it. Moreover, integrating Big Data from multi-omics studies provides the potential to explore the pathophysiological mechanisms of the entire biological continuum of AD. In this context, Artificial Intelligence (AI) offers a wide variety of methods to analyze large and complex data in order to improve knowledge in the AD field. In this review, we focus on recent findings and future challenges for AI in AD research. In particular, we discuss the use of Computer-Aided Diagnosis tools for AD diagnosis and the use of AI to potentially support clinical practices for the prediction of individual risk of AD conversion as well as patient stratification in order to finally develop effective and personalized therapies.

## 1. Introduction

Alzheimer’s disease (AD) is an irreversible neurodegenerative disease that progressively destroys cognitive skills, up to the development of dementia. The clinical diagnosis of AD is based on the presence of objective cognitive deficits (which are, typically, prominent memory impairments). In some cases, AD may show atypical presentations, with impairments in non-amnesic domains (i.e., attention, executive functions, visuo-constructive practice and language) [1]. However, AD shares many common clinical features with other neurodegenerative dementia, including Lewy body dementia [2], frontotemporal disorders [3], and vascular dementia, making early and differential diagnosis difficult, especially in the first stage of the disease [4,5]. In atypical AD, clinical signs of fluent and non-fluent progressive aphasia, or dysexecutive/behavioral changes, may overlap with frontotemporal dementia syndromes [6]; posterior cortical atrophy (PCA) with underlying AD etiology may clinically overlap with dementia with Lewy bodies or corticobasal syndrome [7]. Finally, the occurrence of co-existing pathologies is a common feature in those cases of neurodegenerative diseases that share a common pathogenic mechanism, consisting of extracellular and/or intracellular insoluble fibril aggregates of abnormal misfolded proteins (e.g., the formation of amyloid plaques, tau tangles, or α-synuclein inclusions). In this context, the system biology approach, which aims at the integration of clinical and multi-omics data, can help to detect and recognize the pathophysiological and molecular changes characteristic of AD or other pathologies, as well as the associated clinical manifestations occurring, in particular, in the pre-clinical stages [8].

Amyloid plaques and neurofibrillary tangles are the neuropathological hallmarks of the disease [9,10], which can be evaluated in vivo by neuroimaging investigation and cerebrospinal fluid (CSF) biomarker assessment; namely, considering amyloidβ1-42 (Aβ42), its ratio with amyloid β1-40 (Aβ42/Aβ40), total tau protein (t-tau), and hyperphosphorylated tau (p-tau181).

As AD is multi-factorial, many conditions can influence the individual risk and age of onset, particularly metabolic impairments (diabetes mellitus, hypertension, obesity, and low HDL cholesterol), depression, hearing loss, traumatic brain injury, and alcohol abuse [11]. Lifestyle factors such as smoking, low physical activity, and social isolation are potentially modifiable, while several of these may have bi-directional relationships and may be early manifestations, other than risk factors, in the prodromal phase of dementia [12]. All of these clinical, biological, socio-demographic, and lifestyle factors contribute to defining the development of the disease and, therefore, are useful in trying to understand the still-misunderstood etiology of AD.

Decades of experimental and clinical research have contributed to unraveling many mechanisms in the pathogenesis of the disease, such as the β-amyloid hypothesis, but the puzzle remains incomplete. Clinical and biological data from electronic health records and multi-omics sciences represent a potentially unlimited amount of information about biological processes, such as genomes, transcriptomes, and proteomes, which can be explored through Big Data exploitation [8,13]. The rapid collection of data from tens of thousands of AD patients far exceeds the human ability to make sense of the disease. 

Complex AI-based models can be successful in extracting meaningful information from Big Data; however, as their complexity increases, it becomes more and more difficult to interpret how they produce their output. Thus, they have been called black-box models. Making AI explainable is a key problem of AI technological development in recent years, and is of pivotal importance in healthcare applications, where both patients and clinicians need to trust research methods to make decisions about people’s health [14]. The AD pathology is characterized by high complexity and heterogeneity, and many authors have demonstrated the absence of etiological uniformity and diverse treatment suitability for each patient, supporting the need for an accurate individual diagnosis [15,16]. In order to make the most of biological experiments and refine their findings, they have to be supported and followed by complex biological modeling, based on mathematical and statistical tools such as Artificial Neural Networks [17]. Formalized domain expertise from psychology, neuroscience, neurology, psychiatry, geriatric medicine, biology, and genetics can be integrated with novel analytic approaches from bioinformatics and statistics to be applied on Big Data in AD research projects, with the aim of answering detailed questions through the use of predictive models. These can succeed in answering key questions about promising biomarker combinations, patient sub-groups, and disease progression, finally leading to the development of effective treatment strategies, helping patients with tailored medical approaches [18].

In this context, AI technology represents a promising approach to investigate the pathological mechanisms of AD by analyzing such complex data. In this review, we focus on recent findings using AI for AD research and future challenges awaiting its application: Will it be possible to make an early diagnosis of AD with AI? Will AI be able to predict conversion from Mild Cognitive Impairment (MCI) to AD dementia, stratify patients, and identify “malignant” forms with worse disease progression? Finally, will AI be able to predict the course and progression of the disease and help in finding a cure for AD?

### 1.1. AI and the Biomedical Research

**Figure 1:**
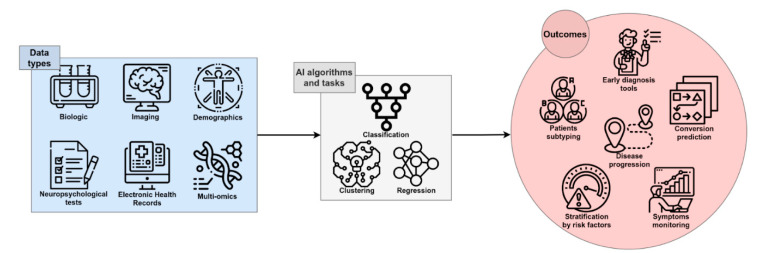
The framework of AI in AD research. Single-modality or integrated data can be processed by several algorithms and tasks, leading to useful outcomes for early and accurate diagnosis, prediction of the course and progression of disease, patient stratification, and discovery of novel therapeutic targets and disease-modifying therapies.

AI has recently significantly revolutionized the way that digital data is analyzed and used. Currently, in some applications, AI is used to perform simple tasks, such as face or speech recognition, and often outperforms human abilities in those tasks [19]. This is definitely a great opportunity to be transferred to medical care, due to the potential for fast, low-cost, and accurate automation; for example, in the processing of digital images by AI algorithms [20]. Several studies have been performed with the attempt to ameliorate the knowledge of complex multifactorial diseases such as AD: AI exploits the features of Machine Learning (ML) and Deep Learning (DL) to develop algorithms that can be used in the clinical and biomedical fields for the classification and stratification of patients, based on the integration and processing of a large variety of available data sources, including neuroimaging, biochemical markers, clinical, and neuropsychological (NPS) data from patients and controls. An extensive application of AI in the biomedical field is for Computer-Aided Diagnosis (CAD). This kind of application aims to automate the diagnostic process upon data analysis, potentially contributing to reaching an early and differential diagnosis of AD or dementia of different etiology (Figure 1).

ML is a fundamental branch of AI, consisting of a collection of data analysis techniques that aim to generate predictive models by learning from data, progressively improving the ability to make predictions through experience (see glossary; Table 1) [21]. DL is a sub-field of ML and uses methods that are able to learn relationships between inputs and outputs by modeling highly non-linear interactions into higher representations, at a more abstract level (see glossary) [22]. Moreover, there are two main categories of predictive models based on ML or DL techniques: Supervised and unsupervised. In supervised learning, the algorithms learn from labeled data to associate an input (e.g., cortical thicknesses data) with a specific output (e.g., presence/absence of a disease or neuropsychological test performance), leading to models that are able to predict the output variable. In unsupervised learning, the algorithms learn from unlabeled data with the purpose of identifying clusters among the observations, based on similar features (see glossary); unlike in supervised learning, there are no correct answers and the aim of the algorithm is to discover structures within variables.

AI techniques have found a wide range of applications in the clinical and biomedical fields, in an effort to automate, standardize, and improve the accuracy of early prediction (regression task), classification of patients (classification task), or the stratification of subjects, based on the processing of specific data (see glossary). In a classification task, the algorithm is trained to associate a label (e.g., “AD diagnosis”) to a given set of features (e.g., clinical parameters, cognitive status, genotypes, biochemical markers, imaging, and so on), in order to be able to generate predictions. Once the model is ready, it is able to predict a class, defined by a label (e.g., “AD” or “control”), by analyzing the set of features of a new given example. The regression task instead involves predicting the value of a variable (e.g., “hippocampus volume” or “biomarker level”) measured on a continuous scale.

Despite ample research effort, we still do not have a cure capable of modifying and/or halting the course of the disease. Some clinical trials are ongoing, especially with the use of monoclonal antibodies targeting Aβ peptides, modified Aβ species, and monomeric as well as aggregated oligomers, which have shown to be safe and have clinical efficacy in AD patients [23]. However, AI pipelines can be applied in automatic compound synthesis in order to analyze the literature and high-throughput compound screening data, to perform an initial molecular screening and automated chemical synthesis [24]. By updating the AI model after cell- or organoid-based experiments, AI can be used to propose a new molecular optimization plan and new bioassays can be conducted to evaluate the biological effects of the compound, thus enabling an automated drug development cycle based on AI design and high-throughput bioassay, greatly accelerating the development of new drugs [25]. AI technology can be used to repurpose known drugs for treatment of Alzheimer’s disease [24,26,27,28]. This is a fast, low-cost drug development pathway, in which AI is used to predict drug repurposing by analyzing large-scale transcriptomics, molecular structure data, and clinical databases. Finally, AI can be exploited to simplify clinical trials too, both in the design and implementation phase [24]. Participant selection can be optimized by using AI algorithms on genetic and clinical data, thus predicting which subset of the population may be sensitive to new drugs [29]. Notably, coupling AI with data from wearables enables almost real-time non-invasive diagnostics, potentially preventing drop-out at subject level [30]. Although promising and rapidly growing, only few of these AI applications have made it to the clinical application stage; nonetheless, AI represents a promising technology to support research and, finally, to develop novel effective therapies [24,31].

### 1.2. Public Databases and Biobanks

The application of AI-based techniques for AD and other diseases research requires extensive data sets, composed of hundreds to thousands of entries describing subjects over many clinical and biological variables, which can be employed to develop novel algorithms by analyzing the features of the disease. In the last 20 years, many open data-sharing initiatives have grown in the field of neurodegenerative disease research [32]; and, in particular, in AD research. Some important data-sharing resources are the Alzheimer’s Disease Genetics Consortium (ADGC, www.adgenetics.org, accessed on 30 May 2021 Date Month Year), Alzheimer’s Disease Sequencing Project (ADSP, www.niagads.org/adsp/content/home accessed on 30 May 2021), Alzheimer’s Disease Neuroimaging Initiative (ADNI, http://adni.loni.usc.edu/ accessed on 30 May 2021), AlzGene (www.alzgene.org accessed on 30 May 2021), Dementias Platform UK (DPUK, https://portal.dementiasplatform.uk/ accessed on 30 May 2021), Genetics of Alzheimer’s Disease Data Storage Site (NIAGADS, www.niagads.org/ accessed on 30 May 2021), Global Alzheimer’s Association Interactive Network (GAAIN, www.gaain.org/ accessed on 30 May 2021), and National Centralized Repository for Alzheimer’s Disease and Related Dementias (NCRAD, https://ncrad.iu.edu accessed on 30 May 2021) [33,34]. Such public databases and repositories collect biological specimens and data from clinical and cognitive tests; lifestyle, neuroimages; genetics; and CSF and blood biomarkers from normal, cognitively impaired, or demented individuals, which can be combined to apply cutting-edge ML algorithms. Moreover, the National Alzheimer’s Coordinating Center (NACC) has constructed a large relational database for both exploratory and explanatory AD research, by use of standardized clinical and neuropathological research data [35]; DementiaBank, the component of TalkBank dedicated to data on language in dementia, provides data sets from verbal tasks such as the Pitt corpus, which contain audio files and text transcriptions from AD subjects and controls [36,37].

In the so-called “*Omics era*”, several databases of omics data—not limited to or specific for neurodegenerative diseases—have been established, such as the Gene Expression Omnibus (GEO), which collects functional genomics data of array- and sequence-based data regarding many physiological and pathological conditions, including AD, among others [38]. Finally, UK Biobank collects and stores healthcare databases and associated biological specimens for a wide range of health-related outcomes from a large prospective study including over 500,000 participants [39].

In the AD field, public and private databases represent the substrate and the source for AI to facilitate a more comprehensive understanding of disease heterogeneity, as well as personalized medicine and drug development.

## 2. AI for AD Diagnosis: Is It Possible to Make an Early Diagnosis of AD with AI?

**Figure 2:**
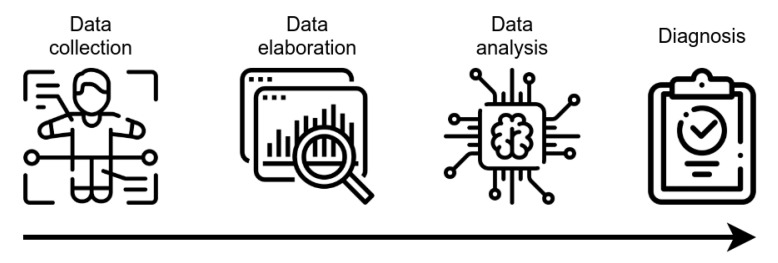
Schematic representation of CAD tools functioning. After collection, data are elaborated, in order to be made ready for the analysis using AI-based techniques. The outcoming result is the assignment of a class, with potential value for diagnostic evaluation.

AI technology, mainly ML algorithms, can handle high-dimensional complex systems that exceed the human capacity of data analysis. ML has been used in the CAD of many pathologies, including AD, by combining electronic medical records, NPS tests, brain imaging, and biological markers, together with data obtained by novel developed tools (e.g., wearable sensors) for the assessment of executive functions (Figure 2). Magnetic Resonance Imaging (MRI), Positron Emission Tomography (PET), 18F-fluorodeoxyglucose-Positron Emission Tomography (FDG-PET), and Diffusion Tensor Imaging (DTI) provide detailed information about the brain structure and functionality, allowing for the identification of features supporting the diagnosis, such as atrophy, amyloid deposition, or microstructural damages [40,41]. Moreover, neuroimages can discriminate pathological processes not due to AD that can lead to cognitive decline (e.g., brain tumors or cerebrovascular disease). Several studies have demonstrated that markers of primary AD pathology (CSF Aβ1-42, total tau and p-tau181, amyloid-PET), neurodegeneration (structural MRI, FDG-PET), or biomarker combinations can be integrated into complex tools for diagnostic or predictive purposes [6,42,43,44]. Of interest, polymorphism in the apolipoprotein E (APOE) gene is the strongest genetic risk factor in the sporadic form of AD, which has an added predictive value, with the APOEε4 allele conferring an increased risk of early age of onset, while the APOEε2 allele confers a decreased risk, relative to the common APOEε3 allele [45].

So far, the first CAD tools for AD were constructed through the use of AI methods for the analysis of brain imaging [46,47]. Analyzing MRI data from the OASIS database [48], a feature extraction and selection method called “eigenbrain”, which is carried out using PCA (see glossary), was used to capture the characteristic changes of anatomical structures between AD and NC; namely, severe atrophy of the cerebral cortex, enlargement of the ventricles, and shrinkage of the hippocampus. The applied SVM algorithm achieved a mean accuracy of 92.36% for an automated classification system of AD diagnosis, based on MRI data [49]. For instance, by using FDG-PET of the brain, a DL algorithm for the early prediction of AD was developed, achieving 82% specificity and 100% sensitivity at an average of 75.8 months prior to the final diagnosis [50].

The majority of ML models for classifying AD from NC are trained with neuroimaging data, which have the advantage of high accuracy [51], but limitations associated to their high cost and lack of diffusion in non-specialized centers. A large group of studies have focused on the identification of fluid marker panels as potential screening tests. In addition to Aβ- and tau-related biomarkers, novel candidate markers according to other mechanisms of AD pathology have been investigated in experimental and meta-analysis studies, in order to optimize the predictive modeling.

A recent study has applied ML algorithms to evaluate data on novel biomarkers that were available on PubMed, Cochrane Systematic Reviews, and Cochrane Collaboration Central Register of Controlled Clinical Trials databases. Experimental or review studies have investigated biomarkers for dementia or AD using ML algorithms, including SVM, logistic regression, random forest, and naïve Bayes. The panel included indices of synaptic dysfunction and loss, neuroinflammation, and neuronal injury (e.g., neurofilament light; NFL). An algorithm, developed by integrating all the data from such fluid biomarkers, has been shown to be capable of accurately predicting AD, thus achieving state-of-the-art results [52].

To the end of designing a blood-based test for identifying AD, the European Medical Information Framework for Alzheimer’s disease biomarker discovery cohort conducted a study using both ML and DL models. Data used for modeling included 883 plasma metabolites assessed in 242 cognitively normal individuals and 115 patients with AD-type dementia, and demonstrated that the panel of plasma markers had good discriminatory power and have the potential to match the AUC of well-established AD CSF biomarkers. Finally, the authors concluded that it can be commonly included in clinical research as part of the diagnostic work-up, with an AUC in the range of 0.85–0.88 [53].

Moreover, AI has the potential to integrate data obtained through the use of new technologies, such as devices designed for the evaluation of language and verbal fluency or executive functions in healthy or mildly impaired individuals. A system for acoustic feature extraction over speech segments in AD patients was developed, by analyzing data from DementiaBank’s Pitt corpus data set [36,37]. The acoustic features of patient speeches have the advantage of being cost-effective and non-invasive, compared to imaging or blood biomarkers, and can be integrated to develop screening tools for MCI and AD. Moreover, another system has exploited a digital pen to record drawing dynamics, which can detect slight signs of mild impairment in asymptomatic individuals [54]. The ability of this pen was evaluated in patients performing the Clock Drawing Test (CDT), which allowed for the identification of subtle to mild cognitive impairment, with an inexpensive and efficient tool having promising clinical and pre-clinical applications.

## 3. Prediction of MCI-to-AD Conversion: Will AI Be Able to Identify Those MCI Subjects Who Will Convert to AD?

Diagnosing probable AD in a subject with moderate–severe cognitive decline or evidence of cortex atrophy is usually not difficult for a skilled neurologist when appropriate data are available. Therefore, it is not surprising for an AI model to solve the task of AD vs. NC subject classification with high accuracy, when taking into account NPS test results or neuroimaging data [55]. To date, several predictive models have been developed [55,56,57,58,59,60,61,62,63,64,65,66,67,68,69,70,71,72], yielding peak accuracy values of 100% in AD vs. NC classification [55]. In contrast, a much more challenging task for AI is to identify individuals with subjective or mild impairment who will develop AD dementia, with respect to stable MCI or MCI not due to AD, given the shaded differences and the overlapping symptoms in the clinical or biological variables defining these groups in the early phases [73].

Algorithms designed to predict MCI-to-AD conversion aim to classify MCI patients into two groups: those who will convert to AD (MCI-c) within a certain time frame (usually 3 years) and those who will not convert (MCI-nc). Yearly, about 15% of MCI patients convert to AD [60,74] and, thus, early and timely identification is crucial, in order to ameliorate the outcome and slow the progression of the disease.

Several AI-based models test the accuracy of combinations of non-invasive predictors, as well as socio-demographic and clinical data, in order to develop effective screening or predictive tools.

By using the ADNI data set, socio-demographic characteristics, clinical scale ratings, and NPS test scores have been used to train different supervised ML algorithms and, finally, develop an ensemble model utilizing them (see glossary). This ensemble learning application demonstrated a high predictive performance, with an AUC of 0.88 in predicting MCI-to-AD conversion [62], and has the advantage of using only non-invasive and easily collectable predictors, rather than neuroimaging or CSF biomarkers, thus enhancing its potential use and diffusion in clinical practice.

**Figure 3:**
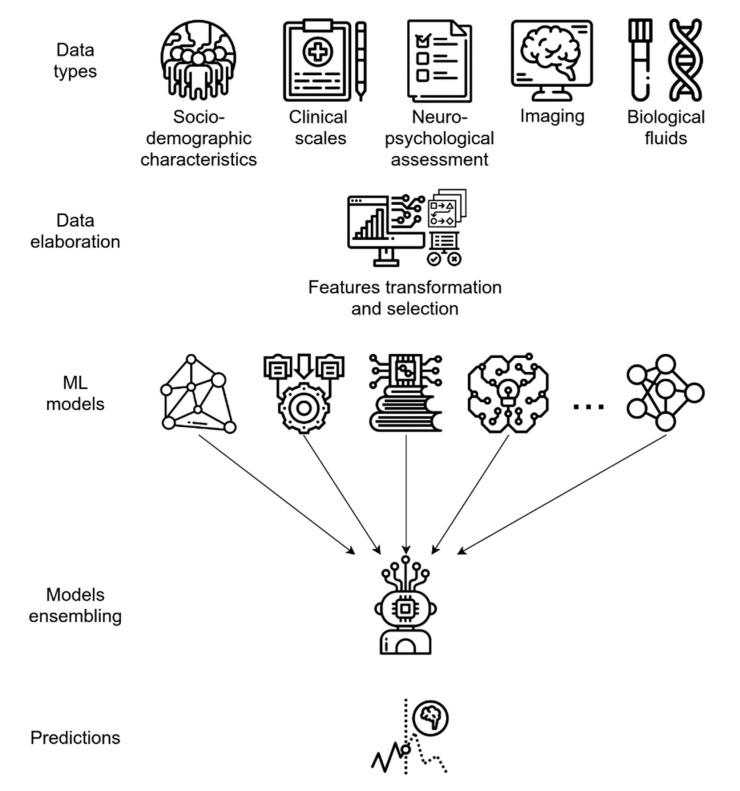
Predictive ML ensemble method for the conversion of MCI to AD based on multi-modal data (i.e., socio-demographics, clinical, NPS, biological fluids, and imaging data). The system uses a feature transformation and selection phase followed by data integration, allowing for more efficient use of variables. The final ensemble of several different ML models provides accurate final predictions of AD or AD conversion.

As for CAD systems, both MRI and PET data can be independently modeled by ML algorithms, yielding good predictive accuracy; however, integrating neuroimaging data with other variables, such as cognitive measures, genetic factors, or biochemical changes, can significatively enhance the model performance, as is generally expected when integrating multi-modal data (Figure 3) [32,64,66,71,72]. For example, the integration of MRI with multiple modality data, such as PET, CSF biomarkers, and genomic data, reached 84.7% accuracy in an MCI-c vs. MCI-nc classification task. When only single-modality data was used, the accuracy of the model was lower than the all-modalities implementation [64].

Different data modalities reflect the AD-related pathological markers that are complementary to each other, which can be concatenated as multi-modal features as input to an ML model for classification [75,76,77]. Notwithstanding, the modality with the larger number of features may weigh more than the others when training the algorithm, inducing bias in the interpretation. In order to overcome this limitation and extract multi-modal feature representations, DL architectures can be used, which do not need feature engineering, due to their ability to non-linearly transform input variables [78].

A DL model for both MCI-to-AD prediction and AD vs. NC classification was trained on data from the ADNI database, including demographic, NPS and genetic data, APOE polymorphism, and MRI. The model processed all the data in a multi-modal feature extraction phase, aiming to combine all data together and obtain a classification output. The AD vs. NC classification task achieved by this model reached performances close to 100%, as expected; whereas, for the MCI-to-AD prediction task, the AUC and accuracy were 0.925 and 86%, respectively [55].

Some models transferred the knowledge in performing AD vs. NC classification to a prediction task—that is, MCI-to-AD conversion—using transfer learning methodology (see glossary). A system with the highest capacity of discriminating AD from NC by analyzing three-dimensional MRI data was recently tested for MCI-to-AD prediction, reaching a high accuracy (82.4%) and AUC (0.83). This finding demonstrated that information from related domains can help AI to solve tasks targeted at the identification of patients at risk of developing AD-related dementia [56].

An interpretation system was embedded with a classification model for both early diagnosis of AD and MCI-to-AD prediction, in order to increase the impact in clinical practice. This model integrates 11 data modalities from ADNI, including NPS tests, neuroimaging, demographics, and electronic health records data (e.g., laboratory blood test, neurological exam, and clinical symptom data). The model outputs a sentence in natural language, explaining the involvement of attributes in the model’s classification output. The model achieved a good performance while balancing the accuracy–interpretability trade-off in both AD classification and MCI-to-AD prediction tasks, allowing for actionable decisions that can enhance physician confidence, contributing to the realization of explainable AI (XAI) in healthcare [79].

Most of the AI models for AD are mainly built on biomarkers such as brain imaging, often with the use of Aβ and tau ligands, Aβ- or tau-PET, as well as biomarkers in CSF, which have high accuracy and predictive value; however, their invasive nature, high cost, and limited availability restrict their use to highly specialized centers [80,81,82,83]. A possible turning point has emerged with the recent development of ultra-sensitive methods for the detection of brain-derived proteins in blood, making it possible to measure NFL [84], Aβ42, and Aβ40 [85,86], and tau and P-tau in plasma [87,88]. The accuracy of plasma P-tau combined with other non-invasive biomarkers for predicting future AD dementia was recently evaluated in patients with mild cognitive symptoms from ADNI and the Swedish BioFINDER cohort, including patients with repeated examinations and clinical assessments over a period of 4 years to ensure a clinical diagnosis (https://biofinder.se/ accessed on 30 May 2021). The prediction included not only the discrimination between progression to AD dementia and stable cognitive symptoms, but also versus progression to other forms of dementia. The accuracy of plasma biomarkers was compared with corresponding markers in CSF, and with the diagnostic prediction of expert physicians in memory clinics, based on the assessment at baseline of extensive clinical assessments, cognitive testing, and structural brain imaging [88]. Plasma P-tau in combination with the other non-invasive markers showed a higher value in predicting AD dementia within 4 years, with respect to clinical-based prediction (AUC of 0.89–0.92 and 0.72, respectively). In addition, the biomarker combination showed similarly high predictive accuracy in both plasma and CSF, making plasma an effective alternative to CSF, thus providing a tool to improve the diagnostic potential in clinical practice [88].

## 4. Patient Stratification: Will AI Be Able to Predict the Course and Progression of the Disease?

The conversion from MCI to AD dementia is a binary diagnostic categorization that does not capture the heterogeneity among patients (e.g., the disease progression and rate of cognitive change within the AD continuum), as it is defined by the National Institute on Aging and Alzheimer’s Association (NIA-AA) framework [44].

The rate of cognitive decline in MCI patients can be predicted by taking into account the changes in multiple longitudinal memory NPS test scores, in order to identify progressive AD patients [89,90,91]. Interestingly, the alteration of mechanisms of cortical plasticity can be evaluated by transcranial magnetic stimulation, to predict the clinical progression to dementia. In fact, prodromal AD (positive CSF biomarkers and absence of dementia) and MCI patients (negative CSF biomarker and absence of dementia) who progress to a dementia state within 3 years have significant impaired Long-Term Potentiation (LTP)-like cortical plasticity, relative to the patients who do not. This highlights the potential of LTP-like cortical plasticity as a predictive biomarker of the clinical progression to dementia in patients with memory impairment at prodromal stages of AD, as identifiable with the new diagnostic criteria based on CSF biomarkers [92].

As the ADNI database offers multi-modal data, different models can be built based on biological data or cognitive variables. The results showed that the model fit better when trained with biological data (coefficient of correlation *r* = −0.68) than with cognitive data (*r* = −0.4). This projection represents individual variability in the rate of future cognitive decline, offering useful information for patient stratification and reducing misclassification risk, thus supporting clinical practice and personalized interventions in a precision medicine approach [93].

Toward the aim of estimating the time to conversion to AD in MCI patients, it has been shown that multi-modal neuroimaging and clinical data can be used, even when the data are incomplete and noisy, due to limitations in their collection. Conventional regression models are unable to address the issues of incomplete data and, thus, cannot perform conversion time prediction. A low-rank matrix completion algorithm (LRMC) was applied to fix data deficiency and obtain a Pearson Correlation Coefficient of 0.665, demonstrating that regression models can also be applied to incomplete data [94].

Moreover, AI may be crucial to identify potential factors that increase the probability of AD conversion. The survival analysis is based on a probability model that aims to predict the probability of AD conversion at different future time points, and not when the conversion will occur (see glossary). Generally, the model can be used to estimate the relationship between risk factors and an outcome, in order to understand their role in potentially preventing or advancing the disease onset [95,96]. By building survival models for different times of AD onset, it is possible to gather information about the contribution of different measures in determining the risk of AD.

Aiming to investigate risk factors in MCI-to-AD conversion, a multivariate Cox proportional hazards regression model was built using ADNI multi-modal data. The model generates Hazard Ratios (HRs) for every variable, indicating the change in risk of progressing to AD per 1 unit change in the corresponding covariate. Analyzing HRs from the model including multi-modality data allowed for the identification of reduced gray matter volume in temporal lobe-related network, as observed from MRI images; low glucose metabolism in the posterior default mode network, as measured by PET scan; and increased scores in dementia rating tests and a positive APOEε4-status as factors having significant effects on the progression from MCI to AD, defining subjects who were more likely to convert [97].

Moreover, this technique may be applied to examine whether specific biomarkers are linked to disease progression, which are useful to monitor and predict the course of the disease over time (e.g., by modeling Cox regression at specific time points: 12, 18, and 24 months). In MCI patients, the analysis at 12 months revealed both brain and ventricular volume, APOEε4 genotype, and memory and executive function test performance as significant predictors for AD progression. The analyses at 18 and 24 months revealed only memory and executive functions as significant predictors instead, whereas only memory remained significant at 36 months. Cognitive measures retain their predictability for a longer time during the disease progression, while MRI measures become less predictive over time [98].

With the aim of establishing a biomarker-based ML model for the prediction of AD-related cognitive decline, autosomal-dominant AD data (including amyloid-PET, FDG-PET, structural MRI, and CSF) were used to estimate years to symptom onset as a proxy of cognitive decline, in order to help derive a prognostic index from increasingly complex biomarker data. The model showed accurate prediction and successful generalization to an independent sample of sporadic AD patients in predicting cognitive decline. These findings demonstrated that biomarker-based ML can be efficiently used to derive meaningful prognostic indices to identify subjects at risk of imminent cognitive decline [99].

## 5. AI for Precision Medicine in AD: Can AI Allow for AD Patients Sub-Typing?

Clinically, AD patients can vary in several features (e.g., disease progression rate and response to pharmacological treatment), making the pathology highly heterogeneous.

Moreover, AD is a highly heterogeneous disease in clinical manifestations, disease progression, biological profiles, and response to pharmacological treatment. Such complexity represents a great issue for physicians in diagnosing the disease and is one of the reasons for the many failures in pharmacological trials. A great challenge for AI is, thus, to integrate data to identify sub-groups of individuals with similar features. In AD patients, the assessment of clinical signs, Mini Mental State Examination, CSF biomarker (amyloid-β42, total-tau, and phospho-tau) levels, and inflammatory indices (serum c-reactive protein, fibrinogen, D-Dimers) could define the profile of frail AD patients, who are usually non-responders to pharmacological treatment and rapidly progressive [100].

**Figure 4:**
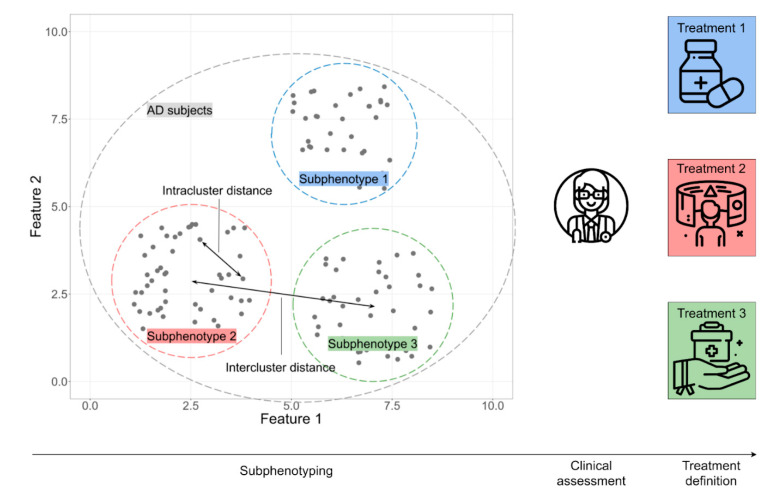
Clustering tools determine groups that share similar properties among populations. Each cluster defines a sub-phenotype with its own peculiar characteristics, providing fine-grained information for the stratification of patients; clustering minimizes the intra-cluster distance, while maximizing the inter-cluster distance. Clinician assessment is pivotal in the development of individually-tailored treatments.

When a huge amount of multi-modal data relative to patients are available, clustering tools, as unsupervised learning applications, can allow for the identification of some sub-groups of individuals that share common characteristics and, hence, improve the development of new targets for tailored treatment strategies. Members within each cluster have high homogeneity for a subset of attribute values, whereas there will be high heterogeneity between different clusters (Figure 4). Evaluation of MCI patients is often noisy, due to the fact that cognitive impairment can have different etiology and manifestations, as defined by multiple biological and clinical factors [94,101,102]. Various applications of unsupervised learning can highlight such complex patterns across subjects or patients, which even an expert clinician may find difficult to discern.

Multi-layer clustering can be used to identify sub-populations among AD or MCI patients [103,104]. A study considering a population of 916 individuals, including 148 AD patients, led to the discrimination of three well-characterized clusters, each defined by its own range of values for both clinical and biological variables, demonstrating that the AD population is non-homogeneous for such factors [104]. When MCI patients were analyzed longitudinally, homogeneous clusters with different prognostic cognitive trajectories were identified; namely, the rapid and slow decliners [103]. The ability of AI to model disease stages in mild AD heterogeneous phenotypes is fundamental for better patient stratification and, thus, for the development of novel effective treatments.

Evaluating differences in clinical characteristics defining clusters of AD patients is a valuable outcome for the application of unsupervised clustering methods. The algorithm “3C” has been used to discover potential clinical biomarkers related to each cluster. Its application to ADNI data has highlighted that subjects can be classified into clusters, showing a greater homogeneity in clinical measurements, compared to those referring to their original ADNI diagnosis [105]. This demonstrates the effectiveness of such an unsupervised learning method in the identification of sub-phenotypic clinical clusters that go beyond current diagnoses, potentially forming homogeneous groups for the enhancement of brain medicine research.

Moreover, unsupervised ML techniques can be applied to identify sub-groups with similar patterns of disease progression. Taking into account not only the phenotypic sub-types, but also the disease stage progression, the application of an algorithm called “SuStaIn” aims to identify AD sub-types and characterize their progression from early to late disease stages. In fact, a probability of sub-type and stage is assigned to each subject, and its application led to the identification of three sub-types among 25 disease stages. Finally, by integrating clinical with imaging data, the algorithm revealed the brain areas from which the brain atrophy originated for each sub-type [106]. Overall, this application provided useful information for in vivo fine-grained stratification of patients, even at early disease stages, and the application of individually tailored biomarker-guided therapies [107,108].

Moreover, integrating data from omics studies holds the potential to explore the pathophysiological mechanisms of the entire biological continuum of AD [8]. Deep Neural Network models can be used to elaborate multiple heterogeneous omics data sets (i.e., involving gene expression and DNA methylation from prefrontal region tissue), representing promising tools to improve the accuracy of distinguishing AD patients by identification of typical features in the biological samples [109]. This application led to the identification of five stable molecular sub-types of AD, which were validated on independent brain RNA data and further characterized by bioinformatic analyses, such as Gene Ontology pathway enrichment and gene co-expression network analysis. These sub-types and their molecular signatures can serve to guide the development of novel therapeutics for AD toward precision medicine [109].

## 6. Future Perspectives

Recently, AI approaches for AD research have shown promising results, providing precise, effective, and convenient methods for assessing AD diagnoses as well as MCI progression in different settings, thanks to the ability to process very large amounts of clinical, biological, environmental, and lifestyle data [51,110,111]. Despite recent progress, AI still faces many challenges, due to the complexity of the disease, for which many mechanisms are still hidden. Similarly, AD is not due to a single genetic or biological component that puts someone at high risk but, rather, to a combination of factors that may define an increased risk. AI can help to manage large data sets that are beyond human capabilities but has some issues that we must take into account. First, we should expect to understand how the algorithm interprets the data and makes decisions: AI should be transparent in order to be trusted by humans, moving in the direction of explainable AI, the purpose of which is to make AI more intelligible [14]. Integrating multi-modal data improves the accuracy of predictions but increases model complexity, making them become non-interpretable (such models are known as black-box models) [112]. To address this issue, feature importance representation techniques, such as SHAP or Grad-CAM, have been developed to explain the decisional processes of black-box models [79,113,114,115]. Second, AI can potentially integrate an infinite amount of data across different modalities, in order to increase the performance of prediction, thus improving their usefulness in clinical practice [116]. Theoretically, increasing the number of features is expected to give more accurate predictions, but continuously joining different data types for multi-modal representation can result in adding irrelevant information, as well as negatively affecting the model performance, if not done correctly. Some data modalities are not well-suited to represent AD patients and, thus, add noise into the subject representation. If the additional features represent misleading information, they can cause overfitting (see glossary) and lead to low performance and generalization ability in the model. Moreover, there is no guarantee that, by increasing the number of data modalities, we will reach 100% accuracy in prediction and, thus, achieve fully representative modeling and a complete understanding of the disease. Therefore, it is possible that some mechanisms of the AD pathology will continue to escape our understanding, even with the application of AI. Finally, AI models need to be objectively evaluated in representative cohorts of patients, in prospective and multi-center validation studies, thus paving the way for translational studies, achieving model fairness, and addressing any source of bias based on gender, ethnicity, or other factors. It is equally important to develop models that improve access to individualized treatment options and provide improved recommendations for AD risk.

The results from most studies presented in this article demonstrated that certain algorithms integrating clinical and biological data may be able to discriminate AD patients, predict AD conversion, or allow for patient sub-typing. Although this evidence is still preliminary, the AI field is rapidly growing and, so, we have reason to believe that AI technology will soon be able to assist in achieving these goals, proposing new hypotheses or theoretical models, and definitively obtaining effective intervention protocols for the disease [24].

## 7. A Framework Overview for Pipeline Architecture in Healthcare

**Figure 5:**
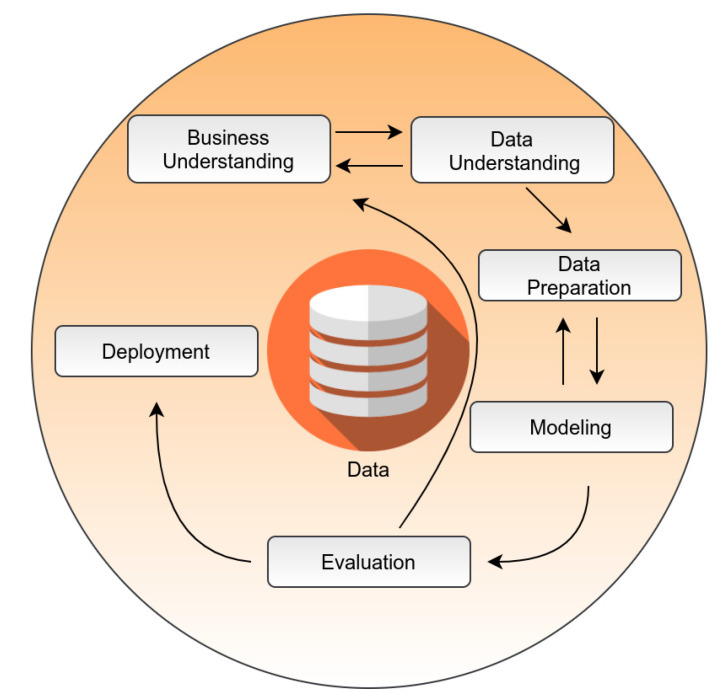
The current cross-industry standard process for data mining (CRISP-DM). It illustrates the six phases of a data mining project, which do not follow a strict sequence. The arrows indicate only the most important and frequent relationships between phases; however, each transition depends on the outcome of each phase, defining the task to be performed next.

AI pipelines are usually deployed to enhance the efforts of clinicians in the prognosis, diagnosis, and drug discovery domains [117]. The no free lunch (NFL) theorem states that no single machine learning algorithm is universally the best-performing algorithm for all problems [118,119]. This also means that we cannot know, in advance, if random forest will be the best-performing algorithm for a classification task on specific data, as there may be others equally or better performing, depending on the data. To overcome this model selection issue, AI pipelines are usually built using the cross-industry standard process for data mining (CRISP-DM) framework, which identifies six major phases in data mining: business understanding, data understanding, data preparation, modeling, evaluation, and deployment [120] (Figure 5). During the modeling phase, various modeling techniques are screened and applied, and their parameters are calibrated to optimal values [120]. Typically, there exist several techniques for the same data mining problem type, where some techniques require specific data formats. All the available algorithms are trained and, in the evaluation phase, the performance of each of these “candidate models” is tested on unseen data, such as cell lines, animal models, or clinical samples in order to see if it can accurately predict the response [121]. Usually, the model that optimally minimizes the cost function—that is, the discrepancy in model performance in testing vs. training steps—is selected for the deployment phase [121]. Deployment is critical, as it consists of a knowledge-transfer phase, where information is delivered to clinicians or researchers for real-world applications. Explainability issues and other constraints in AI application should be treated before the training phase, as requirements are usually defined in the business and data understanding phase of the CRISP-DM routine. CRISP-DM-based pipelines require a large amount of effort by human AI experts, given the NFL theorem, while deployment requires a continuous interaction between healthcare researchers and data scientists. 

## 8. Conclusions

At present, AI models are optimized to find relationships between different data modalities, in order to identify the patterns that predict AD diagnosis and progression and to distinguish between several sub-types of the disease. We expect that future AI models will integrate heterogeneous data to improve the associated robustness and accuracy and will rely on innovations in non-invasive screening tests.

## Figures and Tables

**Table 1:** Glossary.

Method	Definition	Details
Machine Learning	A collection of data analysis techniques that aim to generate predictive models by learning from data and improving their ability to make predictions through experience.	ML models are considered shallow learners, working on data with hand-crafted features defined through expert-based knowledge. Raw data must be pre-processed before constructing a ML system, requiring domain expertise to proceed with feature extraction and engineering, in order to train the algorithm appropriately. As an example of a ML algorithm, a Support Vector Machine (SVM) accomplishes the classification task by finding the hyperplane that, in the multi-dimensional feature space, optimally separates the data into two (or more) classes.
Deep Learning	A sub-field of ML that uses methods that are able to learn relationships between inputs and outputs by modeling highly non-linear interactions.	DL models are different from shallow learners and can elaborate raw data, thus requiring little or no feature engineering, thanks to their ability to model complex functions and identify relevant aspects in the data distribution. DL algorithms are based on Artificial Neural Networks (ANNs), which are inspired by the human brain and can model very complex functions, identifying important aspects in the features and suppressing irrelevant ones. As an example of a DL algorithm, a Convolutional Neural Network (CNN) is composed of nodes organized into layers. It can take an image as input, elaborate the features of the image through its layers, and assign a class attribution as output, thus differentiating between two or more groups.
Supervised learning	A ML task defined through the use of labeled data sets for algorithm training.	The algorithms learn to give the right answer, as defined by the ground truth set, which has labels assigned to the data.An SVM performing the classification task is an example of an algorithm trained by supervised learning.
Unsupervised learning	A set of algorithms aimed to discover hidden patterns or data groupings without the need for human intervention.	In unsupervised learning, unlike supervised learning, there are no correct answers and the algorithm’s aim is to discover structures within variables. The algorithms work with unlabeled data.Common unsupervised methods include clustering algorithms and Principal Component Analysis (PCA).
Classification task	The algorithm is trained to predict a class label.	A classical example is the classification of patients affected by a disease vs. normal controls. The algorithm learns to associate input data with an output label in a supervised manner, and its results can be evaluated by metrics such as accuracy score.
Regression task	The algorithm is trained to predict the value of a continuous variable.	An example is the prediction of hippocampus volume as a numerical quantity. The algorithm learns to associate input data with an output value in a supervised manner, and its results can be evaluated by metrics such as the Root Mean Squared Error (RMSE).
Clustering	Clustering consists of partitioning a data set, in order to find a grouping of the data points.	Clustering is one of the most important unsupervised learning techniques. Its main goal is to reveal sub-groups within heterogeneous data, in such a way that greater homogeneity is shown within clusters (rather than between clusters). Clustering algorithms can lead to the identification of patterns across subjects or patients that are difficult to find even for an expert clinician.
Overfitting	Overfitting occurs when the model is too dependent on training data to make accurate predictions on test data.	When a model is overfitted, its learned ability to separate between two classes does not generalize well to data it has never seen before, therefore limiting its usability for real-world applications.
Ensemble learning	Model ensembling consists of combining multiple ML models, in order to obtain better predictive performance than any of the constituents alone.	A single model alone can be weak in generating predictions. Combining multiple models can compensate for their individual weaknesses.
Transfer learning	A supervised learning technique in which the knowledge previously acquired from the model in one task is used to solve related ones.	In a transfer learning approach, the model is first pre-trained on a source task, then re-trained and tested on a target task. The source task should be related to the target task, with similar relations between the input and output data. In fact, in the pre-training phase, the model gains helpful knowledge for the target task.
Cox regression	The Cox proportional hazards model is a regression technique for investigating the association between the time of an event occurring and one or more predictor variables.	Cox regression gives hazard rates as measures of how factors influence the risk for an event occurrence (outcome), be it death or infection.

## Data Availability

No new data were created or analyzed in this study. Data sharing is not applicable to this article.
